# Targeted Topical Delivery of Retinoids in the Management of Acne Vulgaris: Current Formulations and Novel Delivery Systems

**DOI:** 10.3390/pharmaceutics11100490

**Published:** 2019-09-24

**Authors:** Gemma Latter, Jeffrey E. Grice, Yousuf Mohammed, Michael S. Roberts, Heather A. E. Benson

**Affiliations:** 1 School of Pharmacy and Biomedical Sciences, Curtin Health Innovation Research Institute, Curtin University, Perth 6845, Australia; gemma.latter.bpharm@gmail.com; 2 Therapeutics Research Group, The University of Queensland Diamantina Institute, School of Medicine, University of Queensland, Translational Research Institute, Brisbane 4109, Australia; jeff.grice@uq.edu.au (J.E.G.); y.mohammed@uq.edu.au (Y.M.); m.roberts@uq.edu.au (M.S.R.); 3 School of Pharmacy and Medical Sciences, University of South Australia, Basil Hetzel Institute for Translational Health Research, Adelaide 5011, Australia

**Keywords:** retinoids, adapalene, tazarotene, tretinoin, nanocarriers, liposomes, microemulsion, acne, skin

## Abstract

Acne vulgaris is a common inflammatory pilosebaceous condition that affects 80–90% of adolescents. Since the introduction of tretinoin over 40 years ago, topical retinoid products have been a mainstay of acne treatment. The retinoids are very effective in addressing multiple aspects of the acne pathology as they are comedolytic and anti-inflammatory, and do not contribute to antibiotic resistance or microbiome disturbance that can be associated with long-term antibiotic therapies that are a common alternative treatment. However, topical retinoids are associated with skin dryness, erythema and pain, and may exacerbate dermatitis or eczema. Thus, there is a clear need to target delivery of the retinoids to the pilosebaceous units to increase efficacy and minimise side effects in surrounding skin tissue. This paper reviews the current marketed topical retinoid products and the research that has been applied to the development of targeted topical delivery systems of retinoids for acne.

## 1. Introduction

**Figure 1:**
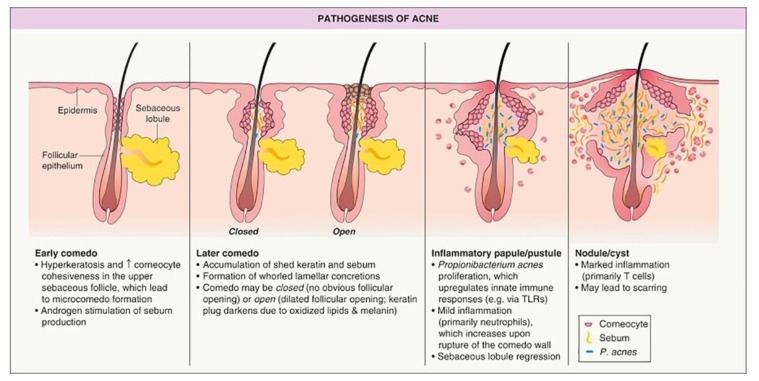
Human hair follicle as a target site for acne treatment showing the pathogenesis of acne: early stage to closed comedo (whitehead) and open comedo (blackhead), papule, pustule and cyst. Reproduced with permission [3], Elsevier, 2012.

Acne vulgaris is a common inflammatory pilosebaceous condition that begins during adolescent years and often persists into adulthood. Epidemiological data suggests that more than 80–90% of adolescents experience acne vulgaris [1,2], with approximately 30% requiring medical treatment. Acne vulgaris presents as lesions including, in order of severity, comedo/comedone, papules, pustules, cysts, inflamed nodules, and can develop into deep, purulent lesions in severe cases (Table 1). The pathogenesis of acne is depicted in Figure 1, taken from Zaenglein and Thiboutot’s chapter on acne vulgaris in the text Dermatology [3], an excellent resource for additional clinical information about the condition. Lesions are most common on the face, but can also present on the back, upper shoulders, neck and chest. Scarring can be a life-long reminder of the condition, and any form of acne vulgaris can cause tremendous detriment to an individual’s mental health and self-image.

Some people continue to get acne well into their adult life. Indeed, “adult-onset acne” is most common in women and is associated with the fluctuating hormones of the menstrual cycle, pregnancy, menopause and starting/stopping the birth-control pill [4,5]. The types of lesions are defined in the following table:

Whilst the precise cause of acne vulgaris is unknown, a number factors are known to contribute to the condition: (i) hyperproliferation of keratinocytes within skin follicles; (ii) androgen-induced hyperproduction of sebum within sebaceous glands; (iii) colonisation of microorganisms such as *Propionibacterium Acnes* (proposed to be reclassified within the new genus *Cutibacterium*) in the excess sebum [6,7]; (iv) inflammation—a factor in all forms of acne, from mild to severe [2,3]. There is also evidence of genetic involvement (a family history of severe acne) [2,3], hormonal factors, environmental and dietary factors, stress and provocation by some medications. The condition is most severe during teenage years due to increasing levels of sex hormones that are converted in the skin to dihydrotestosterone (DHT) which stimulates sebaceous glands to enlarge. These activated sebaceous glands, termed sebocytes, produce increased sebum and proinflammatory factors including lipid peroxides, cytokines, peptidases and neuropeptides. This inflammation contributes to blockages of the follicles so that sebum and dead keratinocytes are trapped to form comedones. Comedone blocked follicles may then rupture leading to increased inflammatory response and so the cycle is perpetuated. The involvement of bacteria within the follicles further stimulates the inflammatory response. Hence this inflammatory cycle is established and requires effective therapeutic intervention.

The initiation of acne occurs when a normal follicle evolves into an invisible subclinical precursor lesion termed a microcomedo, which progresses to a non-inflammatory comedo/comedone and inflammatory lesion (papule, pustule or nodule), as summarised in Figure 1 and defined in Table 1. Recent evidence has suggested that inflammation plays a role in microcomedo formation even before keratinocyte hyperproliferation [8].

A grading system ranging from Grade I (*mild*—presence of non-inflammatory comedones with few inflammatory papules and pustules) to Grade 4 (*severe*—many large papules, pustules and cysts that show widespread inflammation; scarring is often present) is used to classify symptoms and aid in the treatment of acne.

## 2. Current Treatment of Acne Vulgaris

Mild acne is generally treated with regular cleansing of the face with a gentle cleanser, and moisturising the skin with a non-comedogenic moisturiser. Various over-the-counter acne products are available containing actives such as azelaic acid (inhibits *P. acnes* and reduces hyperkeratinisation within follicles), or benzoyl peroxide (keratolytic and weak antimicrobial agent). However, these initial treatments are often insufficient and medical treatment is sought. In Australia, the Therapeutic Guidelines for Dermatology [9] outlines treatment protocols involving a combination of topical retinoid and other topical or oral medication:

- Mild Acne: First line treatment should consist of a topical retinoid—adapalene, isotretinoin, tretinoin, or tazarotene. Gel formulations are recommended for patients with oily skin, while creams are recommended for those with dry or sensitive skin. If minimal improvement is seen after 6 weeks of topical retinoid treatment, add topical benzoyl peroxide, clindamycin or erythromycin.
- Moderate Acne: A topical retinoid or benzoyl peroxide (BPO) is to be applied as per the mild acne guideline, and the strength of the preparation may be increased. It is also recommended that an oral antibiotic is added, such as minocycline, doxycycline or erythromycin. For female patients, hormonal contraceptive therapy may also be added to regulate androgen production.
- Severe Acne: Topical tretinoin is the preferred retinoid to use in moderate to severe cases, combined with one of the aforementioned antibiotics at a higher strength. If still unresponsive, oral isotretinoin therapy is the drug of choice for severe cystic acne.

These guidelines are in line with the ‘Global Alliance on Improving Outcomes in Acne’ which also recommends topical retinoids as first line therapy for acne vulgaris either as monotherapy, or in combination with benzoyl peroxide and/or antimicrobial therapy, depending on severity [10,11]. Clearly, topical retinoids have a central role in the treatment of acne and are therefore the focus of this review, in particular the potential to target their delivery to improve therapeutic outcomes.

There are also a number of physical modalities and complementary therapies that are used in the management of acne [12]. These include chemical peels, laser therapy, plant and mineral-based remedies, as summarised by Fox et al. [12], but beyond the scope of this review.

It is also important to also acknowledge the emerging science on the skin microbiome, its role in acne and potential new therapeutic opportunities [6,7]. Many of the current treatment options directly or indirectly affect the skin microbes including topical and oral antibiotics and retinoids. Their effect on the skin microbiome may not be beneficial to the management of acne. As our understanding of the skin microbiome increases, it is leading to innovative directions that may lead to new therapeutic options. O’Neill and Gallo [6] recently reviewed the microbiome-based approaches to acne treatment that are currently in development. It is likely that we will see microbiome-focused therapeutics emerge in the future that will add to the options for acne management. 

## 3. Topical Retinoids: Efficacy, Safety and Tolerability

Three topical retinoids are currently approved by the U.S. Food and Drug Administration (FDA): adapalene, tazarotene and tretinoin. Topical isotretinoin is available in other jurisdictions. The retinoids are a class of vitamin A-derived medications with multiple mechanisms of action including: decreasing cellular proliferation and differentiation, thereby decreasing the growth rate of follicular keratinocytes; and inhibiting the blockage of follicles and formation of new lesions, including comedones, inflammatory and noninflammatory lesions [13]. It is also thought that the anti-inflammatory mechanism of retinoids is in part due to reducing the release of proinflammatory cytokines [13,14]. Kolli et al. [15] recently provided a systematic review of the clinical study literature on topical retinoids in acne vulgaris. They concluded that the topical retinoids are generally safe and efficacious for the treatment of acne, but also showed that all had common adverse effects of irritation, erythema and dryness. In trials, 62% of patients experienced adverse effects with tretinoin 0.05% gel, 40% with adapalene 0.3% gel and 55.4% with tazarotene 0.05% gel applied daily [16,17]. Irritation associated with topical acne treatments often results in poor patient adherence [18]. Irritancy of topical retinoid products has been studied extensively and novel formulations have been explored to reduce irritancy. In general, topical tretinoin and adapalene preparations were found to be better tolerated than a combination of tretinoin and an antibiotic such as erythromycin [19]. In a well-powered 170 subject study, tretinoin gel microsphere was less irritant compared to adapalene and BPO combination [20]. Another clinical trial showed that the irritancy of tretinoin is directly associated with the concentration used, with higher concentrations being more irritant [21]. Veraldi et.al proposed short contact therapy with tretinoin cream to reduce irritation. Seventy-four patients were treated with 0.05% tretinoin cream once daily for 30 min for a duration of 8 to 32 weeks. Although the efficacy of tretinoin was identical to conventional therapy, irritancy was reduced hence improving the overall adherence to therapy. 

Stability of retinoids is also an issue in development of topical formulations. They exhibit high chemical instability in the presence of oxygen and acids, and photo-instability in the presence of light [22]. Whilst the advice to apply in the evening, thus reducing UV exposure, is sensible, it would be better to develop topical formulations that protect the retinoid drugs both in the product during storage and use.

Currently available topical formulations comprise creams, gels, foams, lotions and solutions in a range of concentrations. In addition, a microsphere-based gel formulation of tretinoin is available. Table 2 provides a summary of the physicochemical properties of retinoids and topical products available.

## 4. Targeted Topical Delivery to the Follicles: A Role for Colloidal Delivery Systems

Humans have approximately 5 million hair follicles, comprised of smaller vellus hair follicles and larger terminal hair follicles [30]. The highest density of vellus hair follicles is on the forehead, with the combined follicular orifices of the face and scalp being 10% of the total surface area, compared to 0.1% at other body sites [31]. The human hair follicle and the sebaceous gland are interconnected by the sebaceous duct, which opens into the follicular duct in the lower infundibulum. Follicular targeting has the potential to selectively treat the site of acne, in particular the hyperproliferation of keratinocytes within skin follicles, and the hyperproduction of sebum within sebaceous glands that supports colonisation of microorganisms such as *P. acnes* and associated inflammation (Figure 1). Lademann et al. [32] have shown that in normal skin follicles may be active, where they are engaged in sebum formation and hair growth, or inactive, where they are not. Penetration into inactive follicles tends to be more limited [32]. This pathophysiology may present a mechanism for targeting to the more active follicles that are secreting the highest level of sebum and are particularly problematic in acne. There is also evidence that some hair follicles are open while others are closed due to being plugged with shed corneocytes and dry sebum [33]. The latter is highly likely for follicles involved in acne. The presence of sebum can restrict permeation into the follicles, particularly for hydrophilic compounds. Sebum is composed of triglycerides (57.5%), wax esters (26%), squalene (12%), cholesterol esters (3%) and cholesterol (1.5%) [34]. In targeting acne, active compounds with more lipophilic properties and thus better solubility in the sebum, have better penetration into the pilosebaceous target areas [35]. Alternatively, formulations capable of interacting with and fluidizing the sebum, such as surfactants and alcohols, could also facilitate delivery to the follicular target sites. However, these formulation properties must be considered in association with the need to ensure that oily products do not exacerbate the underlying problem of oily skin and components such as alcohols and surfactants do not irritate the skin.

An important consideration is the effect of the acne condition on the follicles and consequently the increased degree of difficulty in delivering drug to these target sites. As illustrated in Figure 1, there is an accumulation of sebum, skin and bacterial cells that block access to the follicle. The work of Lademann’s group and others has been focused on understanding follicular delivery in normal skin but acne-involved follicles are entirely different as delivery sites. Nonetheless, there are a number of useful experimental models including comparative skin models (skin plus follicles permeability versus skin without follicles permeability), differential biopsy techniques (skin plus follicle content versus skin without follicle content; for example cyanoacrylate casting) and direct visualization techniques (microscopy, histochemistry, autoradiography), that have been well described elsewhere [36]. 

## 5. Targeted Topical Delivery of Retinoids: Strategies to Improve Efficacy, Safety and Tolerability

There is a substantial body of evidence that shows topically applied colloidal particles preferentially accumulate in the hair follicles and sebaceous glands. Early evidence was provided by a study of TiO_2_ particles applied in a topical sunscreen twice daily for two weeks, in which particles that were detected in the deeper layers of the stratum corneum were associated with the hair follicles [37]. Subsequent research has improved our understanding of the mechanism of follicular targeting, and how the properties of the applied formulation and the method of administration affect delivery. Particle size affects the depth of nanoparticle penetration, particularly where the topical formulation has been massaged on the skin [38]. Lademann’s group suggested that the optimal size of approximately 600 nm corresponds to the cuticle thickness, so that the cuticular layers on the hair shaft that result from keratinocyte desquamation cause the moving hair to act like a geared pump that delivers the nanoparticles into the hair follicles, a process they termed the “ratchet effect” [39]. 

**Figure 2:**
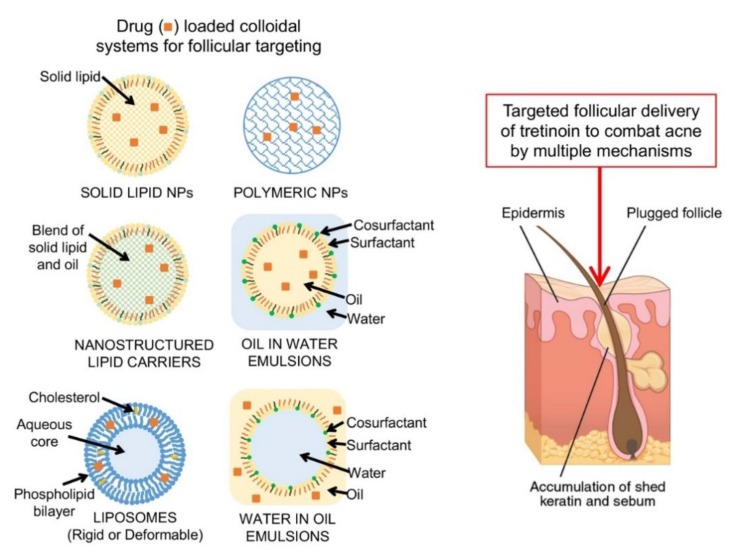
Nanosystems evaluated for the targeted delivery of retinoids to the skin tissues and follicles.

A wide range of colloidal delivery systems have been developed and investigated for targeted topical delivery, including polymeric and solid lipid nanoparticles, nanostructured lipid carriers, liposomes and vesicles, and nanoemulsions and microemulsions [40,41] (Figure 2). These systems offer not just the potential to target the follicles and sebaceous glands, but can potentially also increase drug stability and facilitate the formulation of lipophilic, poorly water soluble drugs. The properties of colloidal systems and their mechanism of targeted delivery are briefly described below.

Polymeric nanoparticles are composed of materials that are generally regarded as safe and efficacious (GRASE). A number of studies have shown that they do not penetrate intact stratum corneum but do accumulate on the skin furrows and hair follicles [42], depending on their properties that can provide sustained release by controlled dissolution, degradation or diffusion [43,44].

Solid lipid nanoparticles (SLN) are composed of lipids that are solid at room temperature and stabilised as a nanodispersion by a surface covering of surfactant [45]. They facilitate the formulation and increase the stability of lipophilic compounds such as retinoids that are prone to decomposition in the presence of light and oxygen [46]. Enhanced follicular targeting is attributed to prolonged contact with the skin surface and interaction between formulation lipids and sebum lipids thereby facilitating permeation of lipid soluble compounds [47].

Nanostructured lipid carriers (NLC) are composed of a fluid lipid phase embedded into a solid lipid matrix or localized at the surface of solid platelets and the surfactant layer [45]. They have a similar targeting mechanism to SLN but the lipids’ spatial structure allows greater drug loading and better stability compared to SLN [48]. 

Flexible liposomes or vesicles are composed of materials that will aggregate into bilayer structures to form spherical vesicles but have the ability to deform in shape. Vesicle compositions include transfersomes (phospholipids with the surfactant sodium cholate), ethosomes (phospholipids with a high proportion of ethanol), niosomes (flexible non-ionic surfactant vesicles), invasomes (phosphatidylcholine, ethanol and a mixture of terpene penetration enhancers), SECosomes (surfactant, ethanol and cholesterol), and PEVs (penetration enhancer containing vesicles) [49].

Nanoemulsions (NE) and microemulsions (ME) are transparent, monophasic, optically isotropic colloidal dispersions composed of oil, water, surfactant and cosurfactant with droplet sizes less than 100 nm and low polydispersity [40,50]. They have high solubilization capacity for both lipophilic and hydrophilic compounds, and their oil and surfactant components facilitate mixing with the skin lipids.

Colloidal delivery systems have been extensively investigated for retinoid delivery, although most research has been based on laboratory-based studies in normal skin in which follicles are more accessible than in acne. There are some examples where these delivery systems have been evaluated in acne-involved skin in small clinical studies with better improvement in acne lesions compared to the commercial topical product [51,52].

In reviewing the approaches to targeted topical delivery of retinoids for acne, it is important to consider that the retinoids are also used in the treatment of psoriasis, and novel delivery approaches targeted to this indication but may also be relevant to acne. Targeted topical delivery of retinoids offers the advantage of concentrating the retinoid at the target site to increase efficacy and reduce the side effects of irritation to other skin tissues. Formulation approaches include the rational design of excipients to enhance thermodynamic activity of the retinoid, provide sustained release and support the skin barrier. In addition, there is a need to address the issue of retinoid stability in the formulation, with colloidal carriers potentially offering enhanced retinoid stability. Another important consideration is that the formulation should provide ease of application and good spreadability, but also good adherence to skin that may be irritated and inflamed due to acne lesions. In general, a semisolid cream or gel is used, with a hydrophilic nature preferred to be suitable for oily skin and alcohol free for minimal irritation. Overall, the aim is to provide a topical product that targets the retinoid to the follicles with minimal systemic delivery, thereby optimising efficacy and minimising adverse effects. It should also be convenient to apply on the skin.

Table 2 summarises much of the current literature available on targeted topical delivery approaches for retinoids, highlighting that the main focus has been on micro and nanotechnology. 

### 5.1. Tretinoin

Tretinoin is highly lipophilic [53] and photolabile, thereby presenting challenges for effective topical formulation. It is a first-generation retinoid, approved by the FDA in 1971 and has been available in cream and gel formulations for many years. It is highly effective but side effects including erythema, peeling, pain and irritation contribute to poor patient compliance. 

A 0.05% tretinoin lotion formulation (Altreno^®^) containing moisturisers was recently approved by the FDA [54]. This formulation consists of micronized tretinoin particles (at least 85% <10 µm) suspended, together with humectants and moisturizers (soluble collagen, sodium hyaluronate, glycerin), in an aqueous structured hydrogel formed by carbomer cross-linked polymers [55]. The tretinoin has limited solubility in the vehicle and is slowly released from the particles, thus optimising the thermodynamic activity of the drug in the formulation and providing a sustained release to the skin tissues. The combination of the sustained tretinoin release and the presence of humectants and moisturisers reduces the irritation and drying effects of tretinoin. The small particle size may also facilitate access to the follicular openings [55]. Deposition of tretinoin in the epidermis and dermis from the tretinoin 0.05% micronized formulation is reported to be 3-fold greater than from the tretinoin microsphere 0.1% gel (21% and 3% compared to 7% and 1% respectively) [56]. Tretinoin photodegradation was significantly reduced when compared to a conventional tretinoin gel (9% and 72% degradation respectively following 8h exposure to UVA light). The lotion was also associated with increased skin hydration and reduced trans epidermal water loss (TEWL) in 30 healthy volunteers, demonstrating enhancement of the skin barrier [55]. The efficacy and tolerability compared to vehicle have been studied in a number of clinical trials with a range of different patient groups [57,58,59,60,61]. Whilst it has not been directly compared to other topical tretinoin formulations in these trials, the incidence of dryness, pain and erythema (4%, 3% and 1% respectively) [57], was lower compared to published experience with other marketed topical tretinoin products (16%, 8% and 7% for a tretinoin 0.05% gel and 12%, 10% and 6% for a microsphere tretinoin 0.1% gel) [62]. This lotion formulation demonstrates how the manipulation of particle size of active and the rational choice of vehicle excipients can be used to enhance clinical outcome in acne.

Retin-A Micro^®^ gel containing tretinoin 0.04%, 0.06%, 0.08% and 0.1% (Bausch Health Companies Inc., Canada) was the first commercially available targeted topical retinoid delivery system. It is based on the Microsponge Delivery System (MDS) [63] with the tretinoin encapsulated in methyl methacrylate/glycol dimethacrylate copolymer porous microspheres (approximately 100–250 nm diameter), within a carbomer-based gel. The principle is that these sponge-like porous, polymeric microspheres will encapsulate and slowly deliver the drug, thereby reducing irritation and increasing stability. One of the earliest applications of the MDS was for the entrapment of benzoyl peroxide, another topical acne treatment that is also prone to irritancy [64]. Wester et al. [64] showed that the MDS provided initial release of BPO for 2 h that was similar to a standard cream formulation, followed by a controlled release over the remaining 6 h period monitored across Silastic membrane in vitro. They attributed the initial release to the BPO on the surface of the microspheres, with the MDS then controlling release. In early studies of the tretinoin MDS gel, efficacy was demonstrated in a multi-centre, double-blind, placebo-controlled 12-week trial of 360 patients, and in a half-face tolerance study of free tretinoin gel verses MDS gel, where 23 out of 25 patients reported less irritancy with the tretinoin MDS gel [63]. Nighland and Grossman [65] conducted a meta-analysis of three randomized, double-blind, vehicle-controlled studies of the tretinoin microsphere gel 0.04% applied once nightly for 12 consecutive weeks in a total of 629 patients. Whilst the MDS tretinoin 0.04% gel was effective in reducing inflammatory and noninflammatory acne lesions, the incidence of adverse effects including erythema, peeling, and dryness in 59.7% to 63.3% was still high. Clearly, there is a need to develop a targeted topical delivery system that can maintain or improve efficacy whist reducing this high incidence of adverse effects. 

Ramenelli et al. [66] recently reviewed the nano delivery system approaches that have been applied to tretinoin including liposomes, niosomes, solid lipid nanoparticles, nanostructured lipid carriers, cyclodextrins and nanostructured polymers. Some examples are described below and other studies are summarized in Table 3 [51,52,67,68,69,70,71]. The earliest work focused on colloidal vesicles including liposomes, niosomes and microsponge/microsphere, which has progressed to a commercially available product, the MDS as described above. Manconi [72] showed that negatively charged niosomes gave better skin retention than those carrying a positive charge, soy phosphatidylcholine liposomes or commercial Retin-A cream. They concluded that tretinoin cutaneous delivery is strongly affected by thermodynamic activity of the drug and vesicle composition (charge, structure and size)—see Table 3 for further details. Follicular deposition and irritation were not investigated, although TEM images of skin showed no evidence of intact vesicles, suggesting that the formulation components were interacting with the skin lipids to facilitate delivery. More recently, Ascenso et al. [73] evaluated ultradeformable vesicles (approximately 150 nm) composed of phosphatidylcholine with Tween 80 as edge activator to confer flexibility. Permeation over 24 h was evaluated in fresh pig ear skin, with tape stripping and remaining skin extractions to determine regional deposition. Tretinoin was predominantly localized in the stratum corneum with no tretinoin detected in the receptor solution. Skin irritation was significantly lower for the vesicles compared to a commercially available tretinoin formulation (Ketrel^®^), as determined by the Draize test following application to the back of shaved Balb/c mice.

A number of studies have shown targeted delivery using nanoparticles of varied compositions [74,75,76,77]. Hydrogels containing lipid core nanoencapsulated tretinoin (composed of poly(ε-caprolactone) polymer, Span 60 and caprylic/capric triglyceride mixture) showed lower tretinoin photodegradation and increased retention in heat separated human epidermis. Ridolfo et al. [75] investigated the addition of chitosan, a complexation polymer with bioadhesion and antibacterial properties, to solid lipid nanoparticles composed of myristyl myristate. Chitosan increased the particle size and conferred antibacterial activity against *P. acnes* and *S. aureus* that could be of potential benefit in acne. They did not evaluate skin distribution, clinical efficacy or irritation. 

Raza et al. [77] prepared and evaluated the physicochemical characteristics of tretinoin-loaded liposomes (phosphatidylcholine, cholesterol), ethosomes (phosphatidylcholine, ethanol), solid lipid nanoparticles (phosphatidylcholine, Tween 80, Compritol 888, ethanol) and nanostructured lipid carriers (phosphatidylcholine, Tween 80, Compritol, ethanol, isopropyl myristate). They then prepared bioadhesive hydrogels of the developed systems and evaluated their photostability, ex vivo permeation and retention in mouse skin and anti-psoriatic activity in a mouse tail model. They reported that the particulate systems provided better photostability, skin transport and anti-psoriatic activity than the vesicle carriers. All nanocarrier systems were more biocompatible and effective than the marketed product (Retin-A^®^ cream).

### 5.2. Tazarotene

Tazarotene is a synthetic retinoid that is available as topical gel, cream and foam formulations containing 0.05% and 0.1% tazarotene under trade names including Tazorac^®^, Zorac^®^ and Avage^®^ (Table 2). Some variability in performance and patient acceptability of these conventional topical formulations has been demonstrated. For example, based on their review of the available literature up to 2016, Smith et al. [78] concluded that the tazarotene 0.1% foam, that was approved by the FDA in 2012, has increased patient compliance and satisfaction compared to other topical tazarotene formulations.

Two groups have examined vesicle-based formulations. Patel et al. [79] reported lower tazarotene permeation and 2–3 times higher skin retention from nanosponge (ethyl cellulose, polyvinyl alcohol and dichloromethane) and noisome (Tween 20, cholesterol and chlororform) gel formulations compared to the commercial Tazorac^®^ formulation. They did not specifically examine follicular deposition or irritation. Proniosomes composed of varying ratios of Span and Tween non-ionic surfactants, and fixed amounts of cholesterol, lecithin, ethanol and phosphate buffered saline containing tazarotene were evaluated [80]. The vesicles ranged in size from approximately 3 to 17 µm and when applied to rat skin over 24 h approximately 40–60% was retained in the skin and 40–60% penetrated to the receptor, with the larger vesicles showing higher retention and lower penetration to the receptor. No irritation was observed when applied to rabbits over 24 h (Draize test). The authors did not compare their formulations to commercial products or examine follicular deposition.

Microemulsions have also been investigated [81,82]. Microemulsions formulations based on 10% Labrafac CC (oil phase), 15% Labrasol and Cremophore-RH 40 (1:1) with 15% Capmul MCM (emulsifiers) and distilled water (external phase), all containing 0.05% tazarotene, were compared with the commercial Tazret^®^ gel (Ranbaxy, Mumbai, India) for rat skin permeation and irritation using the Draize test [81]. Whilst the microemulsion and commercial gel formulations showed similar permeation across rat skin, the amount of tazarotene retained in the skin was significantly higher from the microemulsion. In addition, the microemulsion showed less irritation at 24, 48 and 72 h.

### 5.3. Adapalene

Adapalene is reported to have the best tolerability of the topical retinoids, although adverse effects of dry skin, peeling and erythema are common [15]. Adapalene is commercially available over the counter as Differin gel, containing 0.1% adapalene (Galderma Laboratories, Lausanne, Switzerland) and on prescription for moderate acne as Epiduo^®^ topical gel (Galderma Laboratories), containing the combination of adapalene and BPO (0.1%/2.5%). It is also available in the more concentrated 0.3%/2.5% form as Epiduo^®^ Forte. Adapalene has physicochemical properties that limit its bioavailability in the skin and appendages, in particular its high lipophilicity and pKa 4.23 (Table 2). Colloidal delivery approaches utilising both lipid and solid-based formulations have been investigated to target adapalene delivery to the pilosebaceous units [83,84,85,86,87,88,89,90,91].

Guo et al. [89] designed pH-responsive polymeric nanoparticles composed of Eudragit^®^ EPO to target the lower pH microenvironment in acne skin. The nanoparticles provided lower adapalene crystallinity and higher delivery to pig stratum corneum in vitro compared to a solution of adapalene in a Transcutol^®^-based control vehicle. They did not assess skin irritancy. Nadal et al. [92] incorporated adapalene into microparticles composed of poly ε-caprolactone, a synthetic polyester that is hydrophobic, biodegradable and biocompatible, showing good physical and release characteristics, but have yet to evaluate these in skin. Sallam et al. used the same polymer to form nanospheres that encapsulate and subsequently provide sustained release of adapalene [91]. The negatively charged, lipophilic nanospheres were dispersed in aqueous gels using hydroxyl propyl methyl cellulose (HPMC) and hyaluronic acid (HA) as gelling agents, thus providing sustained release of adapalene. When applied to human skin mounted in Franz cells, the deposition of adapalene from the HA gel formulation was higher than the HPMC gel, nanospheres aqueous dispersion or a commercial gel. Confocal laser scanning microscopy (CLSM) of FITC-labelled nanospheres showed that nanospheres were localized on the stratum corneum and follicles. The nanosphere formulation also proved superior to the marketed adapalene gel in irritation studies using human fibroblasts in cell culture and a 7-day exposure Draize test in rabbits. Of the two test gels, HA gel had a better irritation profile than HPMC-based gel.

Ramezanli et al. [86] also developed polymeric nanocarriers, based on tyrosine-derived 65 nm sized nanospheres (TyroSpheres) that encapsulated adapalene, enhancing its aqueous solubility and reducing crystallinity. The Tyrospheres delivered a significantly higher amount of adapalene to human cadaver epidermis than Differin^®^, with minimal delivery to the dermis, and also significantly higher accumulation in the hair follicles of pig ear skin. Importantly, they also showed that in vitro irritation was significantly reduced in monolayer HaCaTs and reconstituted human epidermis (EpiDerm). They subsequently developed this into a gel formulation and tested the in vivo comedolytic activity in a rhino mouse model, showing that the TyroSphere gel formulation significantly reduced the size of open utricles compared to Differin gel [93]. Decreased secretion of the pro-inflammatory cytokines IL-1α and IL-8, confirmed that the TyroSphere formulation of adapalene was less irritant than Differin.

Kandekar et al. [84] incorporated adapalene into 20 nm diameter spherical micelles composed of d-α-tocopheryl polyethylene glycol succinate deblock copolymer. Adapalene deposition in both porcine ear skin and human skin in vitro was similar from micelle solution and gel formulations and Differin gel and cream formulations, although the dose of adapalene applied was 5 times higher in the commercial formulations. No adapalene permeated to the receptor solution over 12 h from any formulation. Extraction of adapalene from 1 mm sections of skin containing hair follicles compared to follicle-free sections showed that the micelle solution and gel formulations provided 4.5- and 3.3-fold higher delivery respectively when follicles were present. Interestingly, the Differin gel and cream also delivered 4- and 2-fold more adapalene to the skin where follicles were present, although the overall delivery was lower when the dose was considered. It is likely that this reflects the highly lipophilic drug being taken up by the sebum, but also shows the additional benefit of the micelle carrier system.

Bhatia et al. [94] developed a microemulsion with oleic acid as the oil phase, Tween 20 and Transcutol as surfactant and cosurfactant and deionised water. They were able to manipulate the targeting to the follicles by increasing water content, showing that as the microemulsion changed from oil-in-water to bi-continuous the amount in the follicles increased 3-fold.

A liposomal gel formulation incorporating both adapalene and BPO was compared to Epiduo [85]. The approximately 250 nm liposomes significantly increased ex vivo dermal bioavailability and in vivo anti-acne efficacy based on papule density, and reduced skin irritation. Bramman et al. [95] have also recently developed liposomal-based nanosystems of BPO and adapalene for follicular targeting. These studies demonstrated the potential utility of nanocarrier systems to effectively target both of these commonly used acne drugs to the pilosebaceous units.

### 5.4. Isotretinoin

Isotretinoin has the same unfavourable physicochemical properties [96] for formulation and local side effects as other retinoids. It is widely used as an effective oral medication for severe acne but has significant systemic side-effects including the potential for teratogenicity [97]. Topical products are available for mild to moderate acne, though not currently approved by the FDA. Colloidal dispersions have been shown to improve photostability, efficacy and irritation [98,99,100]. Raza et al. [99] prepared isotretinoin-loaded solid lipid nanoparticles using a range of materials and selected the optimum formulation based on entrapment efficiency, skin retention and permeation. The optimal SLN composed of Compritol 888 and phosphatidylcholine were approximately 75 nm, and showed enhanced anti-acne efficacy (testosterone induced acne model in male mice) and tolerability (based on histopathology following administration to female mice) compared to a marketed isotretinoin gel (Sotret^®^ gel, Ranbaxy, India).

An alternate approach involved controlled heating of the skin to enhance isotretinoin solubility and skin delivery [101]. Heat is known to increase skin delivery but may also improve follicular delivery by lowering the viscosity of sebum within the pilosebaceous units. The effect of heat was found to be highly dependent on the vehicle, with a 1:1 binary system of ethanol:propylene glycol monolaurate type II chosen based on delivery to the skin tissue (stratum corneum, epidermis and dermis) and through human skin in vitro. A saturated solution of isotretinoin (1.38% *w*/*v*) in this vehicle was applied to human skin and the follicular deposition determined by cyanoacrylate casting after 1 h, together with skin tissue and receptor amounts. Localised heat (30 min at ≈45 °C) delivered 42% of the applied dose in total, with the heat significantly enhancing delivery to the follicles, epidermis, dermis and receptor (2.3-, 2.1-, 3.2- and 4.2-fold respectively) but not the stratum corneum. The authors concluded that as isotretinoin delivery was increased to the hair follicles and deeper skin layers, but not to the stratum corneum, this provides strong evidence that the enhancement in delivery was via the hair follicles. Thus, the combination of controlled heat and suitable vehicle could target delivery to the pilosebaceous units.

## 6. Conclusions

Whilst there is variability in the efficacy and irritation between the retinoids and the conventional topical formulations in which they are applied, there remains room for improvement by targeted delivery to the follicles and pilosebaceous units. Research has focused on colloidal delivery, with improvements in efficacy and irritation demonstrated using a range of approaches. However, what has received little focus is the method of application. Lademann’s group have clearly demonstrated the benefit of massage of nanoparticles and the importance of size relative to the hair follicle structure for optimising follicular delivery in normal skin. It is not known if massage would also be of benefit for delivery in acne-involved skin, but this should be investigated. Indeed, all laboratory-based studies have used normal skin, with some utilising animal membranes that are not directly comparable to human skin. Even those using human and pig skin are based on normal skin whereas in acne-involved skin the follicles will be plugged with sebum, bacteria and cells and are also affected by inflammation. This is likely to greatly impact access of a dosage form to the follicle. Nonetheless, some clinical studies in patients with active acne have demonstrated improved outcomes with colloidal delivery systems of retinoids showing that this approach is valid even if the laboratory-based models are not entirely appropriate.

It is likely that retinoids will remain the mainstay of acne treatment well into the future, with novel targeted topical formulations that improve efficacy and irritation becoming commercially available. It is also likely that they will be part of the broader suite of treatments that emerge from our developing understanding of the skin microbiome and its role in acne.

## Figures and Tables

**Table 1:** Definitions of lesions associated with Acne vulgaris [2].

Type of Lesion	Definition
Comedone	Keratin-filled plugs that present superficially on the skin. They can be characterised as open or closed. Closed Comedone—commonly known as whiteheads Open Comedone—commonly known as blackheads to due to their dark appearance caused by oxidation of the keratin plug
Papule	A small, raised lesion that is solid to touch and does not contain fluid.
Pustule	A pus-filled lesion caused by increased follicular inflammation and accumulation of inflammatory cells.
Cyst	A deeper skin lesion characterised by an enclosed dilated follicle filled with keratin.
Nodule	A progression from a cyst, caused by destruction of the integrity of the follicle wall and further inflammation; resulting in a large, solid lesion filled with keratin and pus.

**Table 2:** Physicochemical data, topical dosage forms and products for retinoid drugs sourced from [23,24,25,26,27].

Retinoid	Molecular Weight (g/mol)	logP	pKa	Solubility	Available Dosage Forms	Examples of Available Products
Tretinoin	300.442	6.3 logP_o/w_ 5.6 ALogP 6.3 XLogP	4.76	Practically insoluble in water (0.025 mg/L at 25 °C), mineral oil and glycerol. Slightly soluble in polyethylene glycol 400 and ethanol.	Cream 0.1%, 0.05%, 0.02%, 0.025% Gel 0.1%, 0.05%, 0.025% Liquid 0.05% Lotion 0.05% Microsphere gel 0.1%, 0.04%, 0.06%, 0.08%	Cream: Retrieve^®^, Stevia-A^®^, Retin-A^®^, Renova^®^, Rejuva-A^®^ Gel: Retin-A^®^, Atralin^®^ Liquid: Retin-A^®^ Lotion: Altreno^®^ Microsphere gel: Retin-A Micro^®^ Range of generic products from multiple manufacturers
Tazarotene	351.464	5.6 logP_o/w_ 4.43 ALogP 4.9 XLogP	1.23	Soluble in water (0.75 mg/L)	Cream 0.1% and 0.05% Gel 0.1% and 0.05% Foam 0.1%	Cream: Zorac^®^, Tazorac^®^, Avage^®^ Gel: Zorac^®^, Tazorac^®^ Foam: Fabior^®^ Range of generic products from multiple manufacturers
Adapalene	412.529	8.6 logP_o/w_ 6.68 ALogP 7.7 XLogP	4.23	Practically insoluble in water (4.01 μg/L)	Cream 0.1% Gel 0.1% and 0.3% Lotion 0.1% Adapalene 0.1% + BPO 2.5% Adapalene 0.3% + BPO 2.5%	Differin^®^ cream, gel and lotion, and Differin XP gel; also Adaferine^®^ and Differine^®^ internationally Range of generic products from multiple manufacturers Epiduo^®^ gel Epiduo Forte^®^ gel Range of generic products
Isotretinoin	300.442	5.66 and 6.3 logP_o/w_ 5.6 ALogP 6.3 XLogP	~4–5	Insoluble in water (0.126 mg/L at 25 °C). Sparingly soluble in alcohol	Gel 0.05% isotretinoin Combination 0.05% isotretinoin + 2% erythromycin	Isotrex^®^ gel Isotrexin^®^ gel

Partition coefficient: experimentally determined between octanol and water (logP_o/w)_, predicted by atom-based calculation ALogP [28] and XLogP [29].

**Table 3:** Summary of research in targeted topical delivery of retinoids.

Formulation Design	Formulation Composition	Methods for Assessing Skin Delivery/Efficacy/Irritation	Physical Characterisation	Delivery/Efficacy/Irritation Outcomes	Reference
Tretinoin					
Nanoemulsion (NanoE), nanosuspension (NanoS)	NanoE: Isopropyl myristate 10% Glycerol 2.26%, Soybean lecithin 1.20% Water 86.5%; Tretinoin 0.035% NanoS: Soybean Lecithin 0.0035%, Water 99.9%; Tretinoin 0.035%	Franz diffusion cells/new born piglet skin, tape stripping method (10 strips).	Particle size and (PI): NanoE: ~175 nm (0.09) initially; ~745 nm (0.69) after 90-day stability test NanoS: Approx. 330 nm (0.25) across study period Zeta potential: NanoE: −69.7 mV NanoS: −53.2 mV	Tretinoin delivery as % applied dose: NanoE: Stratum corneum: 6.51 ± 0.82 Epidermis: 4.34 ± 1.12 Dermis: 2.26 ± 1.35 Receptor: 6.64 ± 1.80 NanoS: Stratum corneum: 6.86 ± 1.10 Epidermis: 3.09 ± 0.81 Dermis: 1.65 ± 1.19 Receptor: 0.28 ± 0.10 Nanosuspension is more suitable for targeted delivery with minimal systemic absorption, and has better photostability.	[74]
Nanoemulsion (NE)	NE: Caprylic/capric triglyceride 10%, Tween 80 3%, water with preservatives; 0.05% tretinoin Commercial cream (identity undisclosed) as control.	Physical and stability characterisation. No release or skin permeation evaluation. Human split-face study: Ten patients, applied NE and marketed cream to each side of the face daily for 6 weeks. Assessments: TEWL, sebum production, lesions counted and porphyrin assessed by Visiopor^®^ PP 34 N camera (indicates presence of *P. acnes*).	Particle size (nm ± SD), PI, zeta potential: 116.2 ± 0.07, 0.105 ± 0.006, −47.1 ± 11.17 mV Stability: after 6 months, no statistical difference in measured parameters (*p* > 0.05).	Significant lesion reduction after 6 weeks of NE use, no significant lesion reduction with marketed tretinoin emulsion. All parameters related to porphyrin production (size, quantity, value of fluorescent spots) were significantly lower in the NE use compared to conventional tretinoin cream.	[68]
Nanoemulsion (NE) and Nanostructured Lipid Carriers (NLC)	NE: isopropyl myristate 10%, polysorbate 80 2%, Water; Tretinoin 0.05% NLC: isopropyl myristate 9%, cetyl alcohol 1%, polysorbate 80 2%, Water; Tretinoin 0.05%	20 human volunteers applied NE and NLC to 2 sites on volar forearm for one week. Hydration, trans-epidermal water loss (TEWL), erythema index and pH were measured. No permeation or efficacy determination.	NE Particle size (nm ± SD); PI; Zeta potential 116.2 ± 1.48; 0.105 ± 0.028; −47.1 ± 5.23 NLC Particle size; PI; Zeta potential 123.3 ± 1.83; 0.098 ± 0.041; −32.8 ± 3.67	No reported side effects. P values for changes in Hydration, TEWL, erythema index and pH, compared to control measurements taken prior to topical administration: NE: 0.646, 0.139, 0.386 and 0.169, respectively NLC: 0.508, 0.051, 0.139 and 0.333, respectively. *p* value comparison between NE and NLC for the 4 factors: 0.066, 0.721, 0.386, and 0.241. No statistically difference between formulations.	[67]
Liposomes	F13: Phospholipid: cholesterol: dicetylphosphate at ratio 9:1:0.01, dispersed in 1% Carbopol 934 gel; 0.025% tretinoin compared to marketed gel (not disclosed)	Formulation development: 16 formulations evaluated for physical characteristics and release profile (no skin permeation profile determined). Optimised formulation (F13) tested for irritancy (applied to 10 human volunteer’s forearms for 6 h) and efficacy (applied to face of 12 patients with severe acne for 4 weeks) in human volunteers.	F13: particle size 318 ± 28 (nm ± SD); PI 0.434; zeta potential −41.2 ± 1.2; E% 73%. Tretinoin release was 46 ± 5.6% over 5 h. Formulation development trends: adding cholesterol decreased particle size and increased entrapment efficiency (E%). Adding dicetylphosphate increased tretinoin release; no effect on particle size or E%.	Irritation: F13 had significantly lower erythema score (0.2 ± 0.42) compared to same strength tretinoin gel without liposomes (1.8 ± 0.67) and marketed gel (1.4 ± 0.31). Clinical efficacy: F13 had significantly (*p* > 0.05) better improvement in total acne lesions at 1, 2 and 3 weeks compared to marketed gel	[52]
Liposomes	PC (Phospholipon^®^90) or hydrogenated PC (Phospholipon^®^90H), cholesterol and tretinoin in a molar ratio of 5:0.6:2 Liposomes were negatively or positively charged by inclusion of dicetylphosphate (DCP: −ve) or stearylamine (SA: +ve) at 2:1 ratio with tretinoin Multilamellar vesicles (MLV) and unilamellar vesicles (ULV: by sonicaltion of MLV). Comparison with three controls: hydroalcoholic solution (WEt), oil solution and Retin-A^®^ cream.	Physical characterisation, tretinoin release (silicone membrane), skin permeation and retention (newborn pig skin) with tape stripping, using Franz diffusion cells TEM of skin to identify liposomes	Particle size (nm ± SD), E% (± SD): MLV P90/SA: 598 ± 67, 71.80 ± 5.1% P90H/SA: 1163 ± 84, 70.01 ± 2.1% P90/DCP: 536 ± 49, 97.32 ± 1.8% P90H/DCP: 993 ± 122, 96.75 ± 2.2% ULV: P90/SA: 297 ± 74, 93.03 ± 1.7% P90H/SA: 205 ± 53, 91.91 ± 1.9% P90/DCP: 293 ± 53, 75.98 ± 2.5% P90H/DCP: 135 ± 56, 78.53 ± 2.6% PI MLV: 0.4 to 0.6; ULV: 0.2 to 0.3	Tretinoin release trends: −ve > +ve; non-hydrogenated > hydrogenated Skin permeation ranking at 9 h: WEt > P90H/SA > Oil > P90H/DCP > Retin-A > P90/SA > P90/DCP Skin retention ranking at 9 h: P90H/DCP ≈ P90/DCP > P90H/SA > P90/SA > WEt ≈ Oil > Retin-A Local accumulation efficiency ranking (LAC: Retention/permeation): P90/DCP > P90H/DCP > P90/SA > P90H/SA > Oil > Retin-A > WEt Conclusion: negative charge better for skin targeting. TEM did not show any presence of liposomes within the skin layers.	[69]
Liposomes, ethosomes, solid lipid nanoparticles (SLN), nanostructured lipid carriers (NLC)	Liposomes: Phosphatidylcholine 400 mg and cholesterol 100 mg, normal saline Ethosomes: Phosphatidylcholine 400 mg, Ethanol ml, normal saline SLN: Phosphatidylcholine 200 mg, Compritol 888 200 mg, Tween 80 1.2 g, Ethanol 0.8 g, water NLC: as SLN except Compritol 888 140 mg, Isopropyl myristate 60 mg All contained tretinoin 0.05%, butyl hydroxy toluene (2.5 mg: antioxidant) and were dispersed in 1.5% Carbopol 934 gel Retin-A^®^ cream as control.	Physical characterisation including photostability and permeation profiles (Franz diffusion cells/ mouse skin with full skin extraction). Skin histology following daily application to mice for 2 weeks and efficacy (anti-psoriatic activity in mouse tail model) following daily application for 3 weeks were evaluated.	Particle size (nm), zeta potential (mV), E% for – Liposomes: 182, 0.67, 65.01 ± 2.31% Ethosomes: 120, −15.6, 76.42 ± 3.92% SLN: 82.3, −20.1, 86.25 ± 4.36% NLC: 79.5, −23.5, 92.13 ± 3.29%	Skin permeation flux (µg·h^−1^ cm^−2^): liposomal gel 8.03 ± 0.50 ethosomal gel 9.17 ± 0.61 SLN gel 10.49 ± 0.99 NLC gel 10.89 ± 0.79 Retin-A gel 6.11 ± 0.09 Skin Retention (%): Liposomal gel 8.12 ± 0.09 Ethosomal gel 3.31 ± 0.11 SLN gel 4.28 ± 0.08 NLC gel 5.62 ± 0.12 Retin-A gel 1.52 ± 0.04 Skin histology at 2 weeks: Liposome, SLN and NLC formulations well tolerated; ethosomal gel some inflammation; Retin-A showed greatest inflammation. Author conclusions: For deep skin disorders (i.e., acne) SLN = NLC = ethosomes >> liposomes. For superficial skin disorders (i.e., psoriasis) liposomes = SLN = NLC >> ethosomes.	[77]
Ultra-deformable vesicles (UDV)	Phosphatidylcholine (PC) and Tween 80 (combined to produce either 15 or 20% lipid solution) Tretinoin 0.05% compared to Ketrel^®^ cream 0.05%	Tretinoin release and permeation profiles (Franz diffusion cells/ fresh pig ear skin with tape stripping). Cytotoxicity study: human keratinocyte HaCaT cell line. Skin irritation: Draize test in mice.	Particle size: 131 ± 10 (nm ± SD) Zeta potential:−5.9 ± 0.6	UDV gave sustained and controlled release. Skin penetration and permeation: Tretinoin predominantly in stratum corneum, less in epidermis/dermis (0.372 and 0.050 µg µg/cm^2^ respectively) with none detected in receptor over 24 h. Cytotoxicity: UDV not cytotoxic at 0.05% tretinoin. Skin irritation: UDV had significantly lower erythema score (<0.5) than marketed formulation.	[73]
Proniosomes	P8: Span 60 and cholesterol at ratio 3:1, sorbitol (1 g/mol total Span/cholesterol content), dispersed in 1% Carbopol 934 gel; Tretinoin 0.025% compared to marketed gel (Acretin gel 0.025%)	Formulation development: 9 formulations evaluated for physical characteristics and release profile (no skin permeation profile determined). Optimised formulation (P8) tested for irritancy (applied to 10 human volunteer’s forearms for 6 h) and efficacy (applied to face of 12 patients with severe acne for 4 weeks) in human volunteers.	P8: particle size 330 ± 46 (nm ± SD); PI 0.46; zeta potential −41.2 ± 1.2; EE 94%. Tretinoin release was 46 ± 5.6% over 5 h. Formulation development trends: particle size increased and E% decreased for Span 40 compared to Span 60, and as cholesterol to surfactant ratio increased.	Skin irritation: P8 had significantly lower erythema score (0.14 ± 0.37) compared to same strength tretinoin gel without niosomes (1.7 ± 0.76) and marketed gel (1.5 ± 0.53). Clinical efficacy: P8 had significantly (*p* > 0.05) better improvement in total acne lesions at all time points up to 4 weeks compared to marketed gel	[51]
Niosomes	MLV and ULV niosomes prepared from octyl-decyl polyglucoside (Oramix CG110^®^) or decyl polyglucoside (Oramix NS10^®^) to compared effect of lipophilicity and with a −ve (DCP) or +ve (SA) charge. All niosomes contained polyoxyethylene (4) lauryl ether (Brij 30^®^) and cholesterol. Liposomes contained PC (P90) and cholesterol. All formulations prepared as saturated tretinoin concentrations and 20% of saturated concentration.	Physical characterisation, tretinoin skin permeation and retention (newborn pig skin) with tape stripping, using Franz diffusion cells	Tretinoin saturated ULV particle size range (nm ± SD): 225 ± 29 to 366 ± 46 and E% 75.98 ± 2.5% to 99.50 ± 0.9% Tretinoin saturated MLV size range (nm ± SD): 536 ± 49 to 923 ± 49 and E% 71.80 ± 5.1% to 98.47 ± 0.8% Tretinoin unsaturated ULV size range (nm ± SD): 87 ± 32 to 229 ± 49 Tretinoin unsaturated MLV size range (nm ± SD): 219 ± 59 to 393 ± 53	For each composition MLV > ULV for tretinoin permeation and ULV > MLV for retention and LAC Tretinoin penetration ranged from 0.221 to 1.104 µg/cm^2^ (NS10/DCP and (CG110/SA) and retention from 12.81 to 79.47 (CG110/DCP and BR30/DCP). LAC ranking: NS10/DCP ≈ BR30/DCP >> NS10/SA ≈ BR30/SA ≈ P90/DCP > P90/SA >CG110/DCP > CG110/SA >Retin-A	[72]
Niosomes (NSV) and liposomes with Labrasol (as PE)	NSV: diolein (Plurol^®^ Oleique CC), cholesterol (5:1) Lab-NSV: diolein, Labrasol (1:1) Liposomes: Phospholipon^®^ 50 Lab-liposomes (Lab-PEV): PC, Labrasol (1.2:1) All formulations 0.25 mg/mL tretinoin	Physical characterisation including stability study (90 days at 4 °C), skin permeation and deposition in stratum corneum (tape stripping), epidermis and dermis using Franz diffusion cells with human abdominal skin. CLSM of fluorescent-labelled vesicles with hydrophilic and lipophilic markers.	Particle size (nm ± SD), Zeta potential (mV), E%: NSV: 156 ± 4, −48 ± 1, 15 ± 9% Lab-NSV: 245 ± 52, −57 ± 2, 79 ± 7% Liposomes: 112 ± 5, −65 ± 5, 96 ± 4% Lab-PEV: 148 ± 18, −82 ± 4, 100 ± 3% Stability: <10% change in all physical measures	Drug deposition in total skin as a % of total applied dose: Liposomes: ~18 Lab-NSVs: ~13 Lab-PEVs: ~9 Diolein-NSVs: ~7 Highest drug deposition was in stratum corneum for all formulations. The addition of labrasol resulted in better drug delivery than addition of diolein. CLSM: Maximum deposition of both markers on stratum corneum surface; lipophilic marker showed no intact vesicles in skin; accumulation of Lab-NSV in hair follicles	[71]
Penetration enhancer containing vesicles (PEV)	PC vesicles with addition of different hydrophilic penetration enhancers: Oramix NS10, Labrasol, Transcutol P, Propylene glycol (PG) Tretinoin cream (pharmacy compounded; undisclosed formulation) as comparator.	Physical characterisation including 90-day stability test, ex vivo skin permeation and “retention” (calculated by difference of amount applied and penetrated), using Franz diffusion cells. Histological examination was performed on excised mouse skin.	Particle size ranged from 125 to 164 nm; Zeta potential from −58 to −69 mV; E% ranged from 82 to 91%. Stability: particle size and zeta potential of vesicles with Labrasol and Transcutol remained constant, NS10 and PG vesicles 40% increase in mean diameter.	Tretinoin penetration was higher for PEV than tretinoin cream and “skin retention” lower or similar; LAC was best for tretinoin cream. Note the methodology for “skin retention” is unreliable. Authors reported better histological outcomes for PEV but methodology unreliable as this was performed on excised skin.	[70]
SLN with and without chitosan	SLN: myristyl myristate as lipid, with/without chitosan	Physical characterisation including stability study (at 4 °C protected from light), cytotoxicity study (HaCaT cell lines exposed for 24 h then analysed by MTT reduction assay), antimicrobial activity (minimum inhibitory concentration [MIC] on).	Particle size (nm ± SD), PI, zeta potential (mV), E% (± SD): SLN-tretinoin: 162.7 ± 1.4, 0.133 ± 0.014, −31.9 ± 2.0mV, 96.8 ± 1.2% SLN-chitosan-tretinoin: 284.8 ± 15.0, 0.376 ± 0.033, 55.9 ± 3.1, 99.6 ± 0.3% Good stability over one year.	Cytotoxicity: SLN-tretinoin caused 28% decrease in cell viability, SLN-chitosan-tretinoin did not cause cytotoxicity. Antimicrobial activity: SLN-tretinoin had no antibacterial activity, SLN-chitosan-tretinoin activity against microorganisms—MIC (in units of chitosan concentration) of 0.04 and 0.3 mg/mL for *P. acnes* and *S. aureus* respectively	[75]
**Tazarotene**					
Microemulsion (ME)	Multiple ME formulations evaluated, MBG8 selected as optimal based on physical and permeation profile. MBG 8: 10% Labrafac CC 15% Labrasol - Cremophor RH 40 (1:1) 15% Capmul MCM 60% water. ME Gel: above with Carbopol 971P NF 2% Comparator: Tazret^®^ gel 0.05%	Physical characterisation and 6-month stability assessment, permeation through excised rat skin in Franz cells with PBS/95% ethanol (7:3) receptor phase with skin extraction at 12 h. Skin irritation (Draize patch test) on rabbits	Physical parameters at day 0 and 6 months: Particle size (nm): 20.01 ± 1.28 and 22.08 ± 1.17 Polydispersity Index: 0.117 ± 0.034 and 0.119 ± 0.042 Zeta potential: −3.66 ± 0.23 mV and −3.50 ± 0.22 mV E%: 99.89 ± 2.66% and 99.79 ± 2.66% Viscosity (cp): 37 ± 0.05 and 38 ± 0.057	The permeation profile of MBG8 and Tazret gel were not statistically different but MBG8 had higher skin retention of tazarotene (47.33 ± 0.82 mg, 9.5% of applied dose, compared to 35.00 ± 1.73 mg, 7% of applied dose). MBG8 gel showed no irritation compared to commercial gel which caused moderate erythema.	[81]
**Adapalene**					
Microemulsion (ME)	ME A: 19% oleic acid 38% Tween 20 38% Transcutol 5% Deionized water ME B: 18% oleic acid 36% Tween 20 36% Transcutol 10% Deionized water ME C: 17% oleic acid 34% Tween 20 34% Transcutol 15% Deionized water ME D: 15% oleic acid 30% Tween 20 30% Transcutol 25% Deionized water Control: solution in oleic acid. All with 0.1% adapalene	Physical characterisation, skin permeation using porcine ear skin on Franz cells with tape stripping (~20 times), follicular casting and extraction from epidermis/dermis. CLSM of ME D distribution in stratum corneum and hair follicles.	Particle size (nm): ME A: 181.09 ± 0.63 ME B: 173.05 ± 1.89 ME C: 144.36 ± 1.01 ME D: 118.01 ± 1.96 ME A, B and C W/O; ME D bi-continuous	Adapalene permeation into stratum corneum and follicles increased with increase in ME water content. ME D had 17-fold greater adapalene follicular deposition than control. CLSM: fluorescence labelled adapalene in ME D penetrated to the base of the hair follicle. The control sample had poor penetration.	[94]
Nanoemulsion (NE)	Multiple formulations evaluated. Optimal Tea tree oil NE gel: 6% Tea tree oil 10% dimethylsulfoxide 20% Tween 80 and Span 80 (75:25) 1% Carbopol 934 0.1% adapalene Comparator: marketed adapalene gel 0.1%; adapalene solution in acetonitrile:THF (5:1)	Physical characterisation including stability Permeation and distribution studies using rat skin and PBS:Acetonitrile:THF (3:10:2) as receptor phase. Skin irritation study: applied to NZ white rabbit skin and observed over 72 h Systemic absorption study in rats: applied once a day for 90 days—adapalene content in blood analysed at 45 and 90 days, liver at 90 days.	Particle size, zeta potential: 105 ± 5 nm, 0.073 mV Stability at room temperature for 2 months: all formulations remained transparent; slight increase in mean droplet diameter.	Permeation and distribution: NE; dermis > epidermis > receptor Marketed product: epidermis > dermis > receptor Adapalene solution: receptor > epidermis > dermis [the solvent used will damage the skin barrier so this is not an appropriate control] The use of shaved rat skin and the Franz cell receptor compartment solution of PBS (pH 5.6):Acetonitrile:THF (3:10:2) raises significant questions regarding the validity of this study. This is not a suitable model for human skin. Irritation: NE did not cause any irritation or erythema. Systemic absorption: none detected.	[83]
Micelles	Micelles composed of d-α-Tocopheryl polyethylene glycol 1000 succinate (TPGS), a water-soluble, nonionic amphiphilic derivative of vitamin E conjugated with polyethylene glycol 1000. Range of concentrations investigated. Optimal formulation: Micelle solution—TPGS 138 mg/mL, adapalene 0.02% Micelle gel—as above with 1.5% sodium carboxymethyl cellulose Comparators: Differin^®^ gel and Differin^®^ cream—both 0.1% (*w*/*w*)	Physical characterisation, stability study of micelle solution and gel at 4 °C for 4 weeks, adapalene delivery into porcine ear skin or human skin in Franz cells over 12 h by extraction from skin and hair follicles, and deposition in hair follicles visualised by CLSM	Particle size <20 nm, PI 0.12 −0.17, E% 87.00 ± 2.31% Stability study: adapalene content and particle size constant over 4 weeks	Delivery (ng/cm^2^) after infinite dose [and finite dose] applied to pig ear skin for 12 h: Micelle solution: 907 ± 244 [230 ± 47] Micelle gel: 951 ± 382 [194 ± 38] Differin gel: 832 ± 183 [498 ± 60] Differin cream: 112 ± 30 [98 ± 39] (Micelle and Differin gel equivalent and significantly more than Differin cream—despite Differin 5x adapalene dose of micelles). Delivery to human skin was statistically equivalent to pig skin data. Delivery to pileosebaceous units in human skin (% of applied dose): Micelle solution: 19.2 ± 6.5% Micelle gel: 18.4 ± 9.7% Differin gel: 5.82 ± 2.32% Differin cream: 2.02 ± 0.64%	[84]
Liposomes	Optimized formulation: Lipid mixture: Phospholipon 90H^®^ 72% and cholesterol 28%, 1% adapalene Comparators: Differin gel; 1% adapalene solution in PEG 400.	Physical characterisation, 3-month stability at 25 °C, 4 °C and −25 °C, in vitro skin permeation and deposition with pig ear skin on Franz cells, followed by tape stripping, cyanoacrylate casting of follicles and skin extraction. CLSM for visualising liposome location.	SLN particle size (nm), E%: 86.66 ± 3.5, 97.01 ± 1.84% Stability: little change in particle size or E% at 4 and −25 °C, but 4x increase in diameter and E% changed from 97% to 85% at 25 °C.	Adapalene permeation (µg/cm^2^) into hair follicles: Liposome formulation: 6.72 ± 0.83 Differin gel: 3.33 ± 0.26 Adapalene solution: 1.62 ± 0.054 Adapalene permeation (µg/cm^2^) into epidermis/dermis: Liposome formulation: 1.75 ± 0.33 Differin gel: negligible Adapalene solution: 0.92 ± 0.26 CLSM: adapalene-loaded liposomes were located in the follicle and associated with the hair shaft	[87]
Solid lipid nanoparticles (SLN)	SLN: tristearin 1%, soya lecithin 0.3%, Tween 80 0.2% with adapalene 0.1%. SLN-gel: SLN dispersed in Carbopol 934 1% aqueous gel. Comparator: adapalene solution in undisclosed solvent.	Physical characterization including release using cellulose dialysis membrane. Skin permeation with rat skin in Franz cells (using 80% (*v*/*v*) methanol in PBS as receptor phase) over 24 h, followed by separation of skin layers. Histopathology of FITC-labelled SLN after 8 h application to shaved abdominal rat skin.	SLN particle size (nm), zeta potential and E%: 148.3 ± 2.5, −12.0 mV, 89.90 ± 1.2% Adapalene release from SLN: fast burst release initially followed by slower, sustained drug release.	Rat skin permeation of adapalene: SLN and SLN-gel: epidermis > dermis > receptor. Adapalene solution: receptor > epidermis > dermis Histopathology: poor quality images show widespread fluorescence. Note that the skin permeation model is problematic: rat skin is much more permeable than human skin and the receptor phase would damage the skin (and there was no integrity testing of skin membrane).	[88]
Polymeric Nanoparticles	Eudragit EPO with adapalene (ratio 5:1), 1% aqueous PVA Comparator adapalene in Transcutol solution	Physical characterisation, drug release and permeation using Franz cells and fresh pig back skin, followed by tape stripping 20 times	Particle size (nm), zeta potential, E%: 125.8 ± 3.5, 18.4 ± 2.9 mV, 3.5 ± 0.3% Nanocarriers were amorphous and adapalene crystallinity decreased upon loading.	Adapalene release: steady state flux through silicone membrane = 6.5 ± 0.6 and 3.9 ± 0.4 μg.cm^−2^·h^−1^ for nanoparticles and Transcutol solution respectively. Equivalent amounts of adapalene in stratum corneum after 24 h.	[89]
Polymeric microparticles	Poly-ε-caprolactone (PCL) microparticles formulated: F0: adapalene 0% Polysorbate 80 0.5 g PCL 2 g F10: adapalene 10% Polysorbate 80 0.5 g Purified water 200 mL PCL 1.8 g Adapalene 0.2 g F20: adapalene 20% Polysorbate 80 0.5 g PCL 1.6 g Adapalene 0.4 g Comparator: physical mixture (PM) of PCL and adapalene at 1:1 ratio	Physical characterization and diffusion across Strat-M membrane with photoacoustic spectroscopy	Particle size (µm), E%: F0: 8 ± 4 µm F10: 8 ± 3 µm, 100.44% F20: 7 ± 3 µm, 99.37% Spherical in shape	Photoacoustic spectroscopy showed that microencapsulation decreased the in vitro transmembrane diffusion of adapalene compared to the physical mixture, with the microparticle formulations predominantly on or in the membrane.	[92]
Polymeric nanoparticles (NS)	PCL-NS: poly-ε-caprolactone (PCL) with adapalene (ratio 10:1), 1% PVA as stabiliser PCL-NS dispersed in 2 aqueous gels: NS-HPMC gel: hydroxypropyl methylcellulose (HPMC) 4% NS-HA gel: sodium hyaluronic acid (HA) 1% Comparator: marketed adapalene 0.1% gel	Physical characterisation, ex vivo skin permeation and distribution with human abdominal skin (single male donor) on Franz diffusion cells over 24 h, followed by tape stripping and separation of epidermis and dermis. CLSM of FITC-NS for skin deposition visualisation. Skin compatibility study: human dermal fibroblasts exposed NS for 48 h. Skin irritation: Draize patch test in rabbits for NS gels and marketed gel application over 7 days.	NS particle size (nm), zeta potential and E%: 107.5 ± 8.19, −13.1 mV, 84.73 ± 1.52% Gels provided sustained release of adapalene over 24 h due to hydrophobic NS and hydrophilic gel matrix.	In vitro skin penetration of adapalene in combined skin tissues: NS-HA gel > NS dispersion > NS-HPMC ≈ marketed gel. Penetration to receptor was minimal for all. Skin compatibility: Cells treated with adapalene alone caused a dose related reduction in cell viability. Cells treated with the adapalene NS caused a lower reduction in cell viability. Skin irritation: Adapalene NS in both gel formulations produced no irritation in the first 3 days, and less irritation than marketed adapalene over 7 days. The HA gel produced less irritation than the HPMC gel.	[91]
NLC	NLC: 1% *w*/*w* tristearin and Labrasol (4:1), 0.3% *w*/*v* phospholipid 90NG, 0.2% *w*/*v* Tween 80 with Adapalene 100 mg NLC-gel: above with 1% Carbopol 934. NLC-AP gel: above. Comparator: adapalene gel 0.1% *w*/*w* (Adapen^®^ gel)	Physical characterisation and skin permeation with rat skin in Franz cells (using 75% (*v*/*v*) methanol and DMF (50:1) in PBS as receptor phase) over 24 h, separation of skin layers.	Particle size 268.3 ± 2.5 nm, zeta potential −16.35 ± 0.21, E% 87.29 ± 1.26%	Adapalene deposition in epidermis and dermis was similar for all NLC formulations and significantly higher than Adapen gel. Note that the skin permeation model is problematic: rat skin is much more permeable than human skin and the receptor phase would damage the skin (and there was no integrity testing of skin membrane).	[90]
**Isotretinoin**					
SLN	Multiple SLN formulations evaluated. SLN optimized formulation: Compritol 888 ATO 674 mg Phospholipon 90G 208.5 mg Tween 80 Isotretinoin 0.05% Comparator: Sotret^®^ gel 0.05% isotretinoin	Physical characterisation and stability at 5, 30 and 65 °C for 12 months, permeation in Franz cells with hairless laca mouse skin, followed by skin extraction. Skin histology following daily application to mice for 2 weeks and efficacy on testosterone induced acne model in mice.	Particle size (nm), PI, zeta potential, E%: 75.3 ± 2.4, 0.139, −22.4 mV, 89.49 ± 4.1% Stability after 12 months: SLN formulation, drug content ranged between 97.45% and 102.13%	Permeation and retention range used for optimisation of SLN: 8.85 to 27.29 µg.cm^−2^·h^−1^ and 4.63 to 22.63 µg·cm^−2^ Skin histology: SLN showed no inflammation; Sotret gel showed inflammation. Testosterone acne model: SLN formulation showed significant improvement in lesion development (*p* < 0.001) compared to marketed formulation.	[99]

Phosphatidyl choline (PC), ultra-deformable vesicles (UDV), poly dispersity index (PI), nanoemulsion (NE), microemulsion (ME), nanostructured lipid carrier (NLC), solid lipid nanoparticle (SLN), entrapment efficiency (E%), trans epidermal water loss (TEWL), Local accumulation efficiency ranking (LAC), propylene glycol (PG), penetration enhancer (PE), confocal laser scanning microscopy (CLSM).
